# Differential Regulatory Role of Pituitary Adenylate Cyclase–Activating Polypeptide in the Serum-Transfer Arthritis Model

**DOI:** 10.1002/art.38772

**Published:** 2014-09-26

**Authors:** Bálint Botz, Kata Bölcskei, László Kereskai, Miklós Kovács, Tamás Németh, Krisztián Szigeti, Ildikó Horváth, Domokos Máthé, Noémi Kovács, Hitoshi Hashimoto, Dóra Reglődi, János Szolcsányi, Erika Pintér, Attila Mócsai, Zsuzsanna Helyes

**Affiliations:** 1 Department of Pharmacology and Pharmacotherapy, University of Pécs Medical School, and Molecular Pharmacology Research Team, János Szentágothai Research Centre, University of PécsPécs, Hungary; 2 Department of Pathology, University of Pécs Medical SchoolPécs, Hungary; 3 Department of Physiology, Semmelweis University School of Medicine, and MTA-SE Lendület Inflammation Physiology Research GroupBudapest, Hungary; 4 Centre for Nanobiotechnology and In Vivo Imaging, Department of Biophysics and Radiation Biology, Semmelweis UniversityBudapest, Hungary; 5 Centre for Nanobiotechnology and In Vivo Imaging, Department of Biophysics and Radiation Biology, Semmelweis University, and CROmed Ltd.Budapest, Hungary; 6 CROmed Ltd.Budapest, Hungary; 7 Graduate School of Pharmaceutical Sciences, Osaka UniversityOsaka, Japan; 8 Department of Anatomy, University of Pécs Medical School, and MTA-PTE PACAP Lendület Research GroupPécs, Hungary; 9 Department of Pharmacology and Pharmacotherapy, University of Pécs Medical SchoolPécs, Hungary

## Abstract

**Objective:**

Pituitary adenylate cyclase–activating polypeptide (PACAP) expressed in capsaicin-sensitive sensory neurons and immune cells has divergent functions in inflammatory and pain processes. This study was undertaken to investigate the involvement of PACAP in a mouse model of rheumatoid arthritis.

**Methods:**

Arthritis was induced in PACAP^−/−^ and wild-type (PACAP^+/+^) mice by K/BxN serum transfer. General features of the disease were investigated by semiquantitative scoring, plethysmometry, and histopathologic analysis. Mechano- and thermonociceptive thresholds and motor functions were also evaluated. Metabolic activity was assessed by positron emission tomography. Bone morphology was measured by in vivo micro–computed tomography, myeloperoxidase activity and superoxide production by bioluminescence imaging with luminol and lucigenin, respectively, and vascular permeability by fluorescent indocyanine green dye study.

**Results:**

PACAP^+/+^ mice developed notable joint swelling, reduced grasping ability, and mechanical (but not thermal) hyperalgesia after K/BxN serum transfer. In PACAP^−/−^ mice clinical scores and edema were significantly reduced, and mechanical hyperalgesia and motor impairment were absent, throughout the 2-week period of observation. Metabolic activity and superoxide production increased in the tibiotarsal joints of wild-type mice but were significantly lower in PACAP^−/−^ animals. Myeloperoxidase activity in the ankle joints of PACAP^−/−^ mice was significantly reduced in the early phase of arthritis, but increased in the late phase. Synovial hyperplasia was also significantly increased, and progressive bone spur formation was observed in PACAP-deficient mice only.

**Conclusion:**

In PACAP-deficient mice with serum-transfer arthritis, joint swelling, vascular leakage, hyperalgesia, and early inflammatory cell accumulation are reduced; in the later phase of the disease, immune cell function and bone neoformation are increased. Elucidation of the underlying pathways of PACAP activity may open promising new avenues for development of therapy in inflammatory arthritis.

Rheumatoid arthritis (RA) is a common inflammatory joint disorder that may severely impair quality of life due to chronic, persistent pain and functional loss. In the early phase it is characterized by edema and tenderness around the affected joints, which are later accompanied by progressive, irreversible degeneration and bone remodeling. Immunologic factors that are predominantly involved in the inflammatory components of RA have been well described. In contrast, the mechanisms of chronic pain and sensitization, as well as the complexity of neurovascular and neuroimmune interactions, have received much less study, although the importance of neurogenic inflammation in the pathophysiologic processes of RA was described long ago (1). The relatively recent availability of biologic therapies has considerably improved the treatment of RA, but early intervention and identification of novel drug targets are imperative to prevent the disease from progressing to the less manageable late phase (2,3).

Pituitary adenylate cyclase–activating polypeptide (PACAP) belongs to the vasoactive intestinal polypeptide (VIP)/secretin/glucagon family (4). It is present in 27– and 38–amino acid–containing forms, the latter being predominant in most mammalian tissues. Its 3 G protein–coupled receptors (GPCRs) can be activated by both forms: PAC_1_ is specific for PACAP, whereas VPAC_1_ and VPAC_2_ are activated by both VIP and PACAP (5). In the last decade it was discovered that PACAP-27 also activates formyl peptide receptor–like 1 (FPRL1), a rhodopsin-like GPCR (6). Activation of the PAC_1_ receptor results in cAMP production, inositol trisphosphate turnover, increased intracellular calcium levels and phospholipase D activity. VPAC_1_/VPAC_2_ receptor activation induces primarily cAMP production (7).

PACAP is widely expressed in the nervous system (e.g., the brain, the superficial layer of the spinal dorsal horn, and capsaicin-sensitive sensory neurons) (7). It regulates nociceptive transmission in a complex manner: it is antihyperalgesic in several peripheral processes, but mainly pronociceptive centrally. Therefore, PACAP has been suggested to play a crucial role in central sensitization and the induction of chronic pain (7–9). We have provided evidence that peripherally administered PACAP-38 is antinociceptive in several models of acute pain, but induces sensitization of the knee joint primary afferents (10). It has been postulated that it activates VPAC_1_/VPAC_2_ receptors located on nociceptive nerve terminals within the articular capsule, thus increasing joint hypersensitivity similarly to VIP, which exerts its pronociceptive effect in osteoarthritis through the same pathway (11). PACAP has a potent vasodilatory effect through the PAC_1_ receptor and facilitates plasma leakage, edema formation, and leukocyte migration (12,13). We have also shown that PACAP has a crucial role in the long-term maintenance of neurogenic vasodilation on the periphery (14).

The role of PACAP in inflammation and immunoregulation has been studied extensively, and it has been suggested to be an important endogenous immunomodulator (7). Its receptors are widely distributed in the immune system: PAC_1_ is expressed on macrophages and monocytes, but not on lymphocytes (15). VPAC_1_ is present on both T and B lymphocytes, macrophages, and monocytes, whereas the VPAC_2_ receptor is expressed only on stimulated lymphocytes and macrophages (16). The FPRL1 receptor is expressed on phagocytic leukocytes, but to a lesser extent also on lymphocytes (17). PACAP-38 induces mast cell degranulation and histamine release, thereby contributing to edema formation (18). In human polymorphonuclear cells it also enhances respiratory burst, elastase, lactoferrin, and matrix metalloproteinase 9 release (19). Interestingly, it stimulates the activity of resting macrophages, but inhibits activated cells (16). PACAP-38 also increases the expression of several neutrophil activation markers, such as CD11b, CD63, and CD66b, suggesting that it has a role in neutrophil-mediated inflammatory pathways (20). Moreover, PACAP-27 specifically induces neutrophil chemotaxis through FPRL1 activation and induces phagocyte activation and Cd11b up-regulation (6). In a model of chronic autoimmune encephalomyelitis PACAP deficiency resulted in increased production of proinflammatory cytokines and chemokines, but decreased synthesis of antiinflammatory cytokines (21).

These data demonstrate a surprisingly pleiotropic effect of PACAP on immune cells. This might be caused by changes in the receptor expression profile during inflammation, as inflammatory stimuli up-regulate several receptors (e.g., FPRL1 or VPAC_1_) and thereby alter the overall effect of PACAP (22).

A possible role of PACAP in bone metabolism has been suggested based on the presence of its receptors in the bone: osteoclasts express PAC_1_, and bone marrow cultures express VPAC_1_, VPAC_2_, and PAC_1_ (23). On desmal osteoblastic cell lines, mainly VPAC_2_ is expressed, and is even up-regulated during the differentiation process. However, little is known about the expression pattern in enchondral bone tissues. Both PACAP-38 and VIP block osteoblast differentiation by inhibiting alkaline phosphatase production and enhance bone resorption by stimulating interleukin-6 production, and thereby, osteoclast-activity (24,25). PACAP also inhibits thyroid hormone–stimulated osteocalcin synthesis in osteoblasts and decreases bone formation (26). It has considerably greater binding and activation at VPAC_2_ receptors on osteblastic cells than does VIP, emphasizing its importance in bone proliferation (27). It has recently been demonstrated that both PACAP-38 and VIP increase the RANKL:osteoprotegerin ratio through activation of VPAC_2_ on osteoblasts, which in turn promotes osteoclastogenesis (28). Despite the above-mentioned reports on the effects of PACAP on bone physiology, there is only one published study describing a chondroprotective effect of PACAP-38 in vitro; this effect was exerted mainly by increasing calcineurin levels (29).

In light of all of these divergent effects of PACAP in inflammatory, vascular, immune, and pain mechanisms as well as in bone turnover, we were interested in its potential role in arthritis. To investigate this we used a model of immune arthritis that has several well-established similarities to RA (30,31).

## Materials and Methods

### Animals and reagents

Experiments were performed on 10–12-week-old PACAP gene–deficient mice on a CD1 background and their wild-type counterparts (PACAP^+/+^) (32). A total of 92 animals were studied.

Because PACAP-27 and PACAP-38 are products of the same exon, the knockout animals lack both. Animals were bred in the Laboratory Animal House of the Department of Pharmacology and Pharmacotherapy at the University of Pécs, and were maintained in an ambient temperature of 24–25°C on a 12-hour light/dark cycle and provided with standard rodent chow and water ad libitum. As there were no differences in the parameters of interest between male and female animals, both sexes were used (except in the increasing temperature hot plate test, in which male mice cannot be used). The studies were approved by the Ethics Committee on Animal Research of the University of Pécs according to the Ethical Code of Animal Experiments (license no. BA 02/2000-2/2012) and complied with the recommendations of the International Association for the Study of Pain. All reagents were obtained from Sigma-Aldrich unless specified otherwise.

### K/BxN serum–transfer arthritis model

Sera obtained from transgene-positive (K/BxN) and -negative (BxN) mice were pooled separately and stored at −80°C as previously described (30,33). Arthritis was induced by intraperitoneal injection of arthritogenic (K/BxN) or control (BxN) serum on day 0. The amount administered was 150 μl for mice used in functional tests, as debilitating joint dysfunction produces gait abnormalities and limits functional nociception measurements. For in vivo imaging and histologic studies a single dose of 300 μl was used, to produce massive joint inflammation and cellular infiltration. For assessment of structural damage, 2 additional 150-μl booster injections were administered on days 10 and 20 to maintain longer-lasting inflammation and mimic the late, degenerative phase.

### Evaluation of disease severity and hind paw edema

Arthritis severity, hyperemia, and paw volume were assessed daily by evaluation for edema and hyperemia (2 classic signs of inflammation) (33) and measurement of hind limb edema by plethysmometry (Ugo Basile) (34). The results were scored on a scale of 0–10 (0–0.5 = intact limb; 10 = most severe changes).

### In vivo fluorescence imaging of vascular leakage

Indocyanine green (ICG) is a Food and Drug Administration–approved fluorescent cyanine near-infrared dye that, upon intravenous injection, rapidly binds to plasma proteins and remains in the healthy vasculature until excreted by the liver. It is suitable for use in imaging inflammatory hypervascularization and capillary leakage in arthritis both in preclinical models (35) and in patients with RA (36). To overcome its rapid clearance and stability problems when in aqueous solutions, non-ionic emulsifiers are used to encapsulate and stabilize ICG in micelles (37) and increase its plasma elimination half-life (38). Therefore, ICG (0.5 mg/kg) dissolved in a 5% (weight/volume) aqueous solution of Kolliphor HS 15, a macrogol-based surfactant (37), was injected intravenously into mice that had been anesthetized by intraperitoneal administration of 50 mg/kg sodium pentobarbital. Imaging (IVIS Lumina II optical imager; PerkinElmer) was performed 5, 10, 20, 30, and 60 minutes postinjection on days 0, 2, and 5, since vascular leakage is an early sign in this arthritis model. Imaging parameters were as follows: auto acquisition time (F-stop) 1, Binning 2, excitation 745 nm, emission filter >800 nm. Data were analyzed using Living Image software (PerkinElmer). Standardized regions of interest (ROIs) were drawn around the ankle joints. A calibrated unit of fluorescence, the radiant efficiency ([photons/second/cm^2^/steradian]/[μW/cm^2^]) originating from the ROIs was used for further analysis.

### Measurements of mechano- and thermonociception

The mechanical hyperalgesia of the hind paw was measured by dynamic plantar esthesiometry (Ugo Basile). Mechanonociceptive threshold was expressed in grams (34). The thermonociceptive threshold was determined using an increasing-temperature hot plate (IITC Life Sciences) heated increasingly from 30°C until the animal either exhibited nocifensive responses (lifting, shaking, or licking either paw) or the preset maximum (53°C) was attained (10). One conditioning and 2 control measurements were performed before arthritis induction.

### Assessment of motor performance and joint function

To determine joint function and grasping ability, mice were placed on a horizontal wire grid, which was then turned over and maintained in this position for 20 seconds or until the animal fell. This is a simple but reliable method for functional analysis in this model (33). Potential impairment of locomotor coordination related to joint rigidity and consequent dysfunction were also studied using an accelerating rotarod (Ugo Basile) and expressed as the time spent on the wheel (14). Arthritis induction was preceded by 3 control measurements.

### In vivo bioluminescence imaging of myeloperoxidase and NADPH oxidase activity

Luminol (5-amino-2,3-dihydro-1,4-phthalazinedione) and lucigenin (bis-N-methylacridinium nitrate) are used to detect reactive oxygen species (ROS). Luminol bioluminescence detects mainly to neutrophil myeloperoxidase (MPO) activity, and lucigenin bioluminescence detects NADPH oxidase activity and extracellular superoxide production of macrophages (39,40). Na-luminol and lucigenin (Santa Cruz Biotechnology) were dissolved in phosphate buffered saline to form 20-mg/ml and 2.5-mg/ml stock solutions, respectively. Anesthetized mice were injected intraperitoneally with 150 mg/kg luminol (days 0, 1, 2, and 4) or 25 mg/kg lucigenin (days 0, 2, 6, and 10). Bioluminescence imaging was performed 10 minutes postinjection using an IVIS Lumina II. Acquisition time was 60 seconds, F-stop 1, Binning 8. Data were analyzed and ROIs were applied; luminescence was expressed as total radiance (photons/second/cm^2^/steradian) within the ROIs.

### In vivo investigation of metabolic activity by positron emission tomography/magnetic resonance imaging (PET/MRI)

In vivo PET/MRI scans were obtained on day 4 using 2-^18^F-2-fluoro-2-deoxy-d-glucose (^18^F-FDG; 4 MBq per animal administered intravenously) and nanoScan PET/MRI (Mediso). The glucose analog ^18^F-FDG exhibits increased uptake kinetics in high-glucose–using cells of inflamed tissue. ^18^F-FDG accumulation adequately reflects inflammatory macrophage activity in acute arthritis, whereas it is less sensitive to neutrophils or T cells. In chronic arthritis, fibroblast proliferation and pannus formation are the main causes of radioisotope accumulation (41). MR images (gradient-echo–external averaging sequence) using a MultiCell imaging bed (Mediso) were overlaid on the PET scans. PET images were reconstructed with a Nucline Tera-Tomo PET algorithm (ordered-subsets expectation-maximization 3-dimensional reconstruction; Mediso) using 300-μm voxels. The standardized uptake values maximum (SUV_max_) of ^18^F-FDG, i.e., the maximum values of the tissue radioactivity concentration divided by (injected activity/body mass) in the ROIs, were calculated in the ankle joints.

### In vivo micro–computed tomography (micro-CT) analysis of the tibiotarsal joint and bone structures

Micro-CT imaging was performed on the same mice at every time point to minimize interindividual differences. The right ankles were repeatedly scanned (days 0, 7, 14, and 28) using the same settings and 17.5-μm voxel size, with a SkyScan 1176 micro-CT (Bruker). After reconstruction of the scans, bone structural changes were analyzed using CT Analyser software. Standardized ROIs were drawn around the periarticular region of the tibia and fibula, as well as the tibiotarsal and tarsometatarsal joints. In these ROIs bone volume (BV; μm^3^) and bone surface (BS; μm^2^) were calculated and expressed as the percentage of the standardized total volume (TV) of the ROI (% BV and BS density).

### Histologic evaluation of joint inflammation

Mice were killed on day 4 or day 28 to investigate acute and chronic alterations. They were transcardially perfused with 4% buffered paraformaldehyde, and ankle joints were fixed in the same buffer, decalcified and dehydrated, embedded in paraffin, sectioned (3–4 μm), and stained with Safranin O. The slides were evaluated semiquantitatively by a pathologist under blinded conditions. Synovial cell proliferation and mononuclear cell infiltration were scored from 0 (normal) to 3 (maximal severity) (34).

### Statistical analysis

Results are expressed as the mean ± SEM. Statistical evaluation was performed using GraphPad Prism. Distribution of the data was tested by D'Agostino-Pearson or Shapiro-Wilk, test depending on the number of values. The significance of ICG imaging, clinical scoring, and plethysmometry results was evaluated by Kruskal-Wallis test, and functional results by repeated-measures analysis of variance (ANOVA). Wire grid performance was evaluated by log rank test, micro-CT results by two-way ANOVA, in vivo bioluminescence and PET imaging results by Student's t-test, and histopathologic scores by Mann-Whitney U test. *P* values less than 0.05 were considered significant.

## Results

### Reduced joint inflammation and edema in PACAP^−/−^ mice

**Figure 1:**
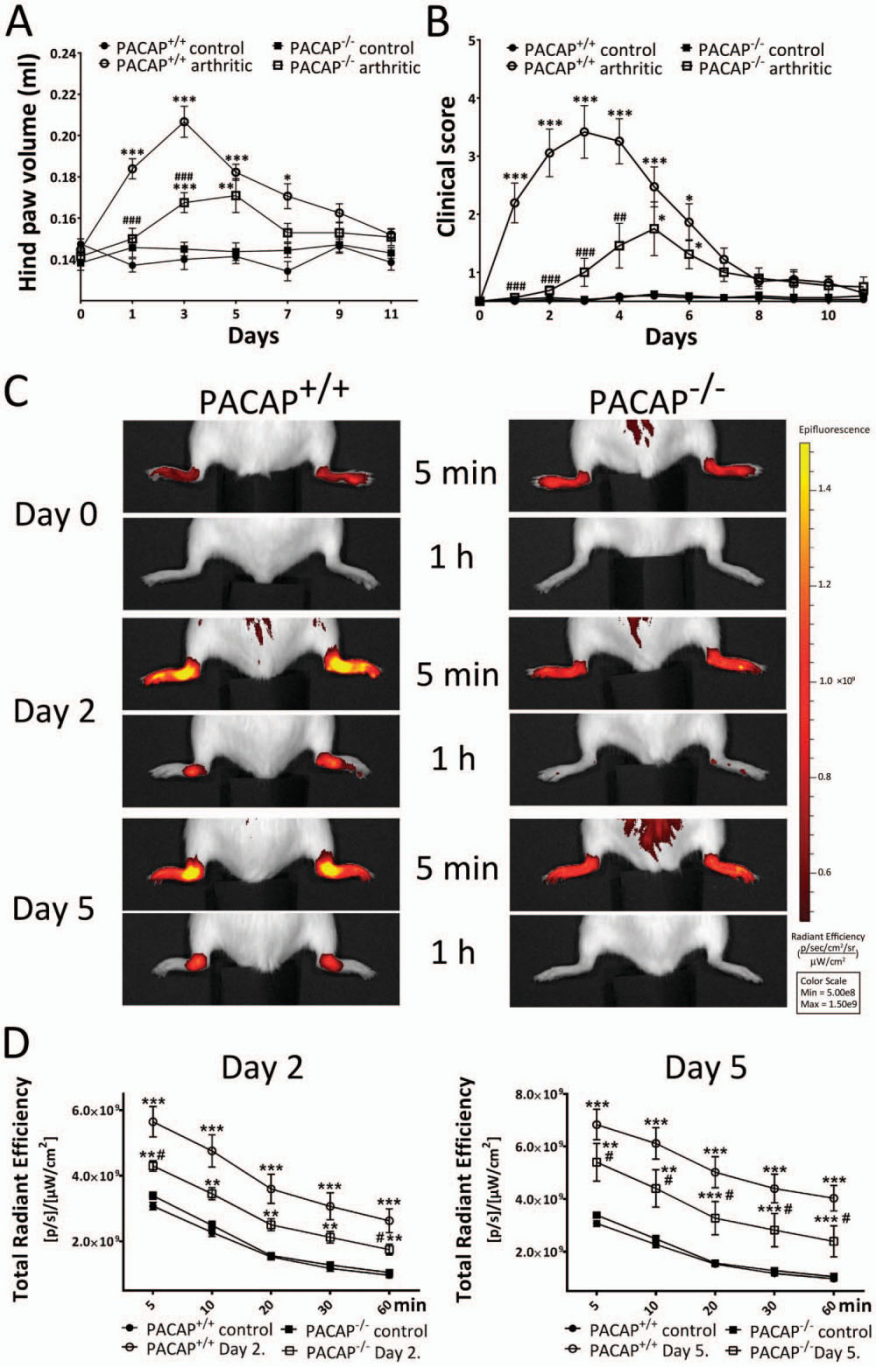
Edema, clinical severity, and inflammatory vascular leakage in PACAP^+/+^ (wild-type) and PACAP^−/−^ mice in which arthritis was not induced (controls administered normal serum) or in which arthritis was induced by administration of K/BxN mouse serum. A and B, Plethysmometric determination of hind paw volumes (A) and clinical scores of disease severity (B) (n = 8−12 per group). C, Representative images of indocyanine green (ICG) fluorescence in the ankle joints, as an indicator of vascular permeability and leakage. Images were obtained 5 minutes and 60 minutes after intravenous injection of ICG (0.5 mg/kg). D, ICG fluorescence intensity ([photons/second/cm^2^/steradian]/[μW/cm^2^] [p/s/μW/cm^2^]) in the ankle joints 2 days and 5 days after induction of arthritis (n = 5−6 per group). Values in A, B, and D are the mean ± SEM. ∗ = *P* < 0.05; ∗∗ = *P* < 0.01; ∗∗∗ = *P* < 0.001 versus the respective control group. # = *P* < 0.05; ## = *P* < 0.01; ### = *P* < 0.001 versus the K/BxN serum−treated wild-type group. Color figure can be viewed in the online issue, which is available at http://onlinelibrary.wiley.com/doi/10.1002/art.38772/abstract.

After induction of arthritis in wild-type mice by administration of 150 μl arthritogenic serum, substantial hind paw edema developed, which peaked on day 3 at 40% and gradually decreased thereafter. In PACAP^−/−^ animals, edema was present to a significantly lesser extent but the kinetics pattern was similar, with the maximum (20%) reached on day 5 (Figure 1A). Semiquantitative clinical scoring of edema and hyperemia showed similar results, but peak scores occurred on day 3−4 in wild-type mice and on day 5 in PACAP^−/−^ mice. Arthritis severity scores were, however, significantly lower in the PACAP-deficient mice (Figure 1B).

### Decreased inflammatory hyperemia and vascular leakage in PACAP^−/−^ mice

Before treatment, ICG fluorescence was similarly low in the ankle joints of PACAP^+/+^ and PACAP^−/−^ mice, demonstrating negligible extravasation of the dye. Two days after induction of arthritis, the accumulation and fluorescence of ICG were notably increased in the ankle joints of wild-type mice both immediately after injection and 1 hour later, indicating hyperemia and plasma leakage (80% increase at 5 minutes, 170% at 1 hour postinjection compared to initial control data). In contrast, in PACAP-deficient animals the increase was significantly smaller (25% at 5 minutes, 65% after 1 hour). By day 5, ICG fluorescence increased even further in both wild-type mice (120% at 5 minutes, 320% after 1 hour) and PACAP^−/−^ mice (60% and 130%, respectively) (Figures 1C and D).

### Diminished mechanical hyperalgesia in PACAP^−/−^ mice

**Figure 2:**
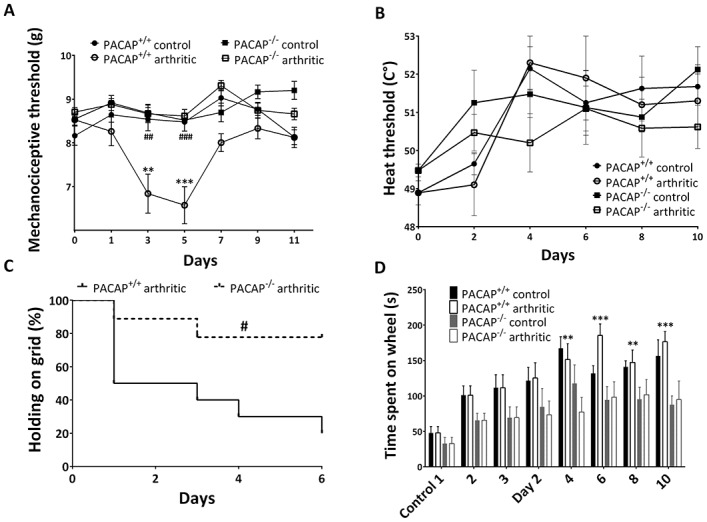
Evaluation of nociceptive changes and motor impairment. A, Mechanonociceptive threshold in the hind paw (n = 8–12 per group). B, Thermonociceptive threshold measured using an increasing-temperature hot plate (n = 4–6 per group). C, Kaplan-Meier curve of the ability to hold onto a wire grid for 20 seconds (n = 8–12 per group). Animals were habituated in 3 control sessions prior to the induction of arthritis. D, Motor performance on an accelerating rotarod during 3 consecutive control measurements and following the induction of arthritis (n = 4–6 per group). Values in A, B, and D are the mean ± SEM. ∗∗ = *P* < 0.01; ∗∗∗ = *P* < 0.001 versus the respective control group. # = *P* < 0.05; ## = *P* < 0.01; ### = *P* < 0.001 versus the K/BxN serum–treated wild-type group.

There was no difference in mechano- or thermonociceptive thresholds between control PACAP^+/+^ and control PACAP^−/−^ mice. In wild-type mice with arthritis, the mechanocociceptive threshold was decreased by 15–20% by day 5 after disease induction, but was normalized by day 9. Mechanical hyperalgesia did not develop in either arthritic PACAP^−/−^ mice or normal serum–treated control PACAP^−/−^ mice (Figure 2A). Arthritis did not result in a significant change in thermonociceptive thresholds in any of the groups (Figure 2B).

### Retained grasping ability of PACAP^−/−^ mice

The horizontal wire grid test revealed an abrupt decrease in grasping ability in wild-type mice; by day 4, only 30% of the mice could stay on the grid for 20 seconds. In contrast, 75–80% of the PACAP^−/−^ animals could stay on the grid for this duration (Figure 2C). Motor performance on the rotarod gradually improved in all groups during the experimental period, demonstrating learning. The improvement was significant in the PACAP^+/+^ group but not in the PACAP-deficent group (Figure 2D).

### Decreased neutrophil MPO activity in the ankle joints of PACAP^−/−^ mice in the early phase of arthritis and increased activity in the later phase

**Figure 3:**
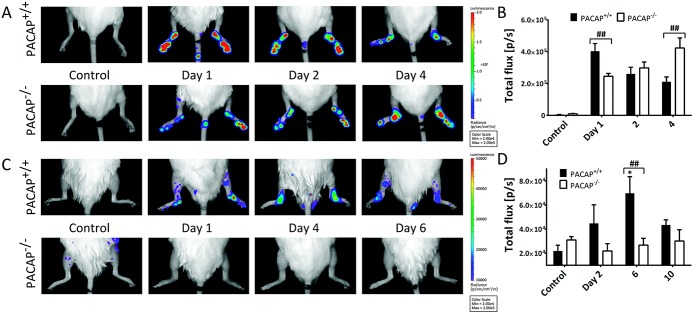
Bioluminescence imaging of neutrophil-derived myeloperoxidase activity and macrophage-derived superoxide activity. A and B, Representative images of luminol activity obtained 10 minutes after intraperitoneal injection of Na-luminol (150 mg/kg) (A) and quantification of luminescence in diseased ankle joints (B). C and D, Representative images of lucigenin-based superoxide detection (C) and quantification of luminescence in diseased ankle joints 10 minutes after intraperitoneal injection of lucigenin (25 mg/kg) (D). Values in B and D are the mean ± SEM photons/second (p/s) (n = 4−6 per group). ∗ = *P* < 0.05 versus control PACAP^+/+^ mice; ## = *P* < 0.01. Color figure can be viewed in the online issue, which is available at http://onlinelibrary.wiley.com/doi/10.1002/art.38772/abstract.

Luminol bioluminescence imaging showing MPO activity in the inflamed joints of PACAP^+/+^ mice peaked in the initial phase of arthritis, reaching a maximum on day 1 and gradually decreasing thereafter. The initially diffuse activity in the hind paws rapidly declined and was concentrated in the tibiotarsal joints by day 4. In contrast, in PACAP^−/−^ mice early MPO activity was significantly lower, but by day 4 it became significantly greater in the ankles (Figures 3A and B).

### Lack of inflammatory superoxide production in PACAP^−/−^ mice

In PACAP^+/+^ mice, lucigenin bioluminescence, indicating the presence of extracellular superoxides, steadily increased, reaching a maximum on day 6. Its elevation occurred more slowly compared to that observed with luminol bioluminescence imaging, highlighting the differences between these markers. Superoxide generation in PACAP^−/−^ mice remained similar to baseline and was significantly lower than that observed in wild-type mice (Figures 3C and D).

### Increased and accelerated periarticular osteophyte formation in PACAP^−/−^ mice

**Figure 4:**
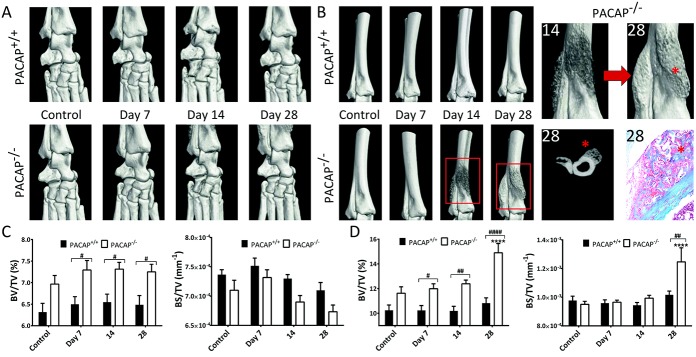
Micro–computed tomography (micro-CT) analysis of structural changes in bone architecture. A and B, Representative 3-dimensional micro-CT reconstruction and histologic analysis of the tibiotarsal joints (A) and the periarticular region of the tibia and fibula (B). In the 4 images on the far right, bone spur formation (asterisks) can be seen in the 3-dimensional reconstructions (magnified views of the boxed areas), in an axial CT slice, and in a photomicrograph of the periarticular region of the tibia and fibula of a PACAP^−/−^ mouse. Original magnification × 100. C and D, Changes in bone volume/total volume (BV/TV) and in bone surface (BS)/TV over time in the vicinity of the tibiotarsal joint (C) and the periarticular region of the tibia (D). Values are the mean ± SEM (n = 6 per group). ∗∗∗∗ = *P* < 0.0001 versus nonarthritic control PACAP^−/−^ mice. # = *P* < 0.05; ## = *P* < 0.01; #### = *P* < 0.0001. Color figure can be viewed in the online issue, which is available at http://onlinelibrary.wiley.com/doi/10.1002/art.38772/abstract.

CT scanning revealed differences in the bone architecture of PACAP^−/−^ mice even in the absence of arthritis. BV/TV in these mice was consistently increased in both the talocrural and distal periarticular regions of the tibia and fibula, although these increases were not significant. Bone surface density in these animals, expressed as BS/TV, was similar to that in wild-type mice. Arthritis did not substantially alter bone characteristics in PACAP^+/+^ mice. In contrast, in PACAP^−/−^ mice it induced extensive, progressive osteophyte formation in the periarticular region of the tibia and fibula, which was apparent by day 14 (Figures 4A and B). These bone spurs had become compact, dense bone by day 28, leading to a prominent, significant increase in bone mass, even reaching 70% extra bone in some mice compared to controls (Figures 4C and D).

### Decreased metabolic activity in the diseased ankle joints of PACAP^−/−^ mice

**Figure 5:**
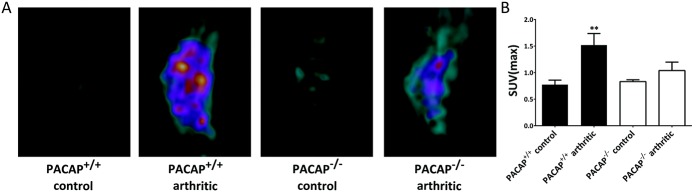
Positron emission tomography/magnetic resonance imaging (PET/MRI) of inflammatory metabolic burst. A, Representative 2-^18^F-2-fluoro-2-deoxy-d-glucose (^18^F-FDG; 4 MBq per animal) PET/MRI multimodal reconstruction images obtained 4 days after induction of arthritis. Areas with increased glucose uptake correspond to the intracapsular space containing tendons and joint capsule internal surface, as identified by both PET and MR signals. B, Quantitative evaluation of the standardized uptake value maximum (SUV_max_) of ^18^F-FDG in the ankle joints. Values are the mean ± SEM (n = 3 per group). ∗∗ = *P* < 0.01 versus nonarthritic control PACAP^+/+^ mice. Color figure can be viewed in the online issue, which is available at http://onlinelibrary.wiley.com/doi/10.1002/art.38772/abstract.

In the inflamed tibiotarsal joints of wild-type mice, metabolism was significantly increased on day 4, as shown by the elevated SUV_max_ values. In contrast, metabolism in the joints of arthritic PACAP-deficient animals did not differ from that observed in nonarthritic controls (Figures 5A and B).

### Increased synovial hyperplasia in PACAP^−/−^ mice

**Figure 6:**
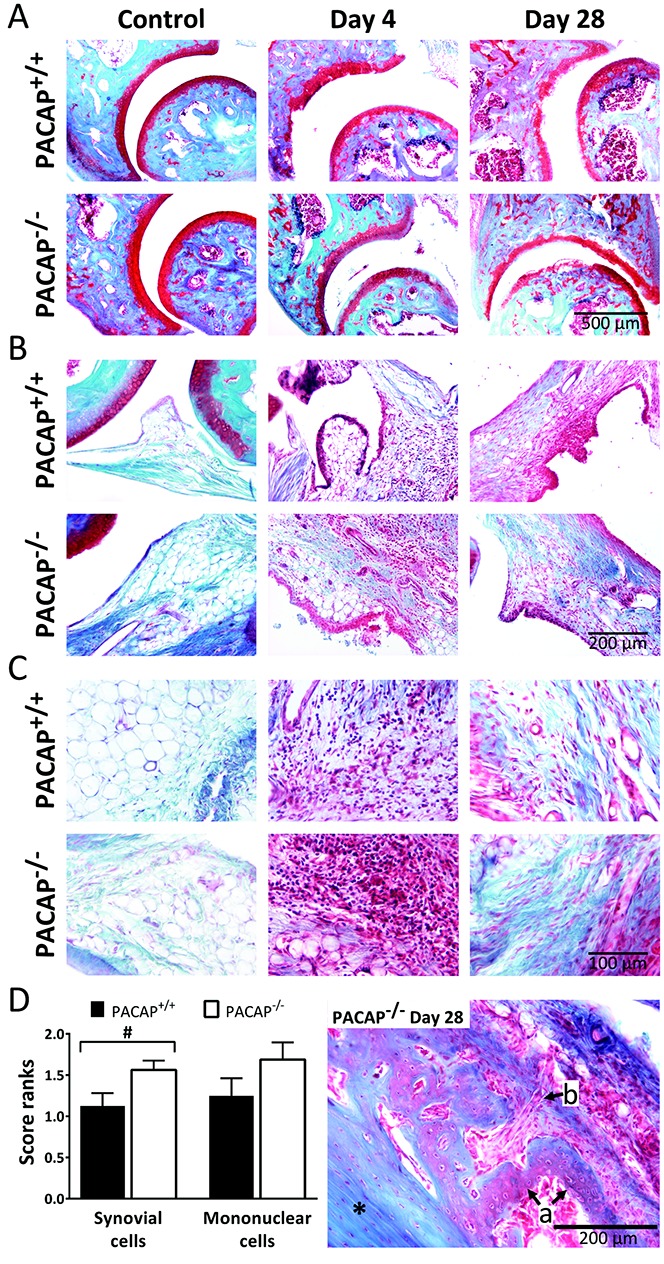
A–C, Representative photomicrographs of the ankle joints of control PACAP^+/+^ and PACAP^−/−^ mice and PACAP^+/+^ and PACAP^−/−^ mice after arthritis induction. Images of the tibiotarsal joints including cartilage lining (A), the synovium (B), and periarticular soft tissue (C) are shown. Blue staining in the day 28 images in B and C represents collagen deposition. Original magnification × 100 in A; × 200 in B and C. D, Left, Semiquantitative histopathologic scores of synovial hyperplasia and mononuclear cell infiltration on day 4. Values are the mean ± SEM (n = 4–6 per group). Right, Representative photomicrograph of a bone spur observed in a PACAP^−/−^ mouse on day 28. Asterisk indicates the tibia. Arrows show irregular, cell-rich new bone (a) and a vessel entering the affected area (b).

Histopathologic analysis of the joints revealed no difference between control serum–treated PACAP^+/+^ and PACAP^−/−^ mice. In both groups the tibiotarsal joint, synovium, and periarticular connective tissue appeared normal (Figures 6A−C). Four days after arthritis induction there were prominent changes in the PACAP^+/+^ group, i.e., an irregular cartilage−bone border, enlarged synovium infiltrated with inflammatory cells, and massive infiltration of immune cells into the periarticular connective tissue with formation of mononuclear cell aggregates (Figure 6A–C). Similar changes were observed in the PACAP^−/−^ mice, and the degree of synovial hyperplasia was greater than that in the wild-type mice whereas the degree of mononuclear cell infiltration was comparable (Figure 6D). By day 28 these acute inflammatory signs had decreased, but the cartilage−bone border became more irregular and the cartilage width was notably reduced in both groups (Figure 6A). The previously infiltrated synovial lining and connective tissue showed prominent collagen deposition, indicating the chronic stage of inflammation. The pathologic spurs in PACAP^−/−^ mice demonstrated on the micro-CT scans were also identified by their irregularity, distinct staining, and notable vascularization seen on histologic analysis (Figure 6D). These features were absent in wild-type mice.

## Discussion

The primary and most important result of our study is the finding, for the first time, of evidence of a surprisingly pleiotropic effect of PACAP on different characteristics in a multifactorial transgenic disease model of RA. K/BxN mice display a spontaneous and progressive polyarthritis, with persistence of high-titer autoantibodies, mainly anti–glucose-6-phosphate isomerase, in the serum (30,31). This serum induces a similar, but transient, arthritis, mainly through inflammatory cytokine production, complement activation, neutrophil activation, and mast cell degranulation.

In this model, which has translational significance, PACAP deficiency decreases vasodilation, plasma leakage, acute inflammatory cell accumulation, hyperalgesia, joint dysfunction, metabolic activity, and ROS generation, while facilitating later neutrophil activity, synovial cell proliferation, and pathologic bone formation. The second important message derived from our study, from a methodologic and translational standpoint, is that the K/BxN serum–transfer model is appropriate for investigating several early and late characteristics of RA using in vivo noninvasive imaging modalities. We adopted and modified in vivo functional and optical imaging techniques, as well as observational longitudinal experimental paradigms that help to identify key pathophysiologic mechanisms in inflammatory and degenerative joint diseases.

The potent edema-forming and acute inflammatory actions of PACAP can be explained by its well-established vasoactive effects (12,42). Our in vivo plasma extravasation imaging results provide deeper insight into the core mechanisms responsible for this phenomenon and validate the functional data. Both VPAC and PAC_1_ receptors are likely to be involved in the vascular effects of PACAP: since nitric oxide might act as an agonist at the VPAC_2_ receptor, its level of synthesis can interact with VPAC-mediated pathways in vasodilation (43), and PAC_1_ receptor is of key importance in the induction of plasma leakage (13,44).

The absence of arthritic hyperalgesia in PACAP^−/−^ mice correlates well with previous results reported by our group and others, demonstrating that PACAP-38 has a pronociceptive role in peripheral pain conditions, in which sensitization mainly occurs in the spinal dorsal horn (8–10,14,45,46). This suggests the importance of PAC_1_ receptor activation in the central nervous system (CNS), since intrathecal administration of a PAC_1_ receptor antagonist had an antinociceptive effect in osteoarthritis (47).

The almost-normal grasping ability of arthritic PACAP^−/−^ mice is likely due to milder joint swelling and pain. We did not find disease-related alterations in motor performance on the rotarod wheel in our experiments, similar to previously reported findings in a model of peripheral neuropathy (14). This can be explained by the fact that this test mainly reflects motor coordination related to CNS processes, and inflammation of the small joints in this model does not influence performance.

We observed an interesting 2-phase pattern regarding the effect of PACAP deficiency on MPO activity during arthritis: in the early phase neutrophil activity is clearly and notably decreased in PACAP^−/−^ mice, correlates well with our functional results but virtually contradicts the traditional view about the inhibitory effects of PACAP on immune cells (16). The explanations are likely to be 1) increased migration of immune cells into the inflamed tissue mainly due to the PACAP-38–driven capillary permeability increase through PAC_1_ receptor activation (12,18,44), and 2) the key importance of PACAP-27 in facilitating neutrophil chemotaxis and migration during the initial phase of the inflammation (6). Thus, we can conclude that these proinflammatory effects of PACAP lead to more rapid formation of the inflammatory microenvironment (19,20).

In contrast, the increased luminol bioluminescence in the later phase suggests that PACAP inhibits neutrophil activity by suppressing proinflammatory cytokine production and increasing the expression of antiinflammatory mediators (16,21). This is consistent with earlier data showing that exogenous administration of PACAP reduces disease severity and inflammatory enzyme production in mice with collagen-induced arthritis (48). However, it is important to note that in the collagen-induced arthritis model there are profound T and B cell responses, whereas K/BxN serum–transfer arthritis depends mainly on myeloid lineages and develops similarly if T and B cells are absent (33). In our model the maximal inflammatory cell activity occurs on day 1 as shown by luminol bioluminescence, which is surprising since this is a very early phase of the disease, when functional changes are minimal. This suggests the importance of further studying this early period to elucidate the mechanisms that precede edema formation and functional loss.

The significantly lower level of extracellular ROS production in gene-deficient mice suggests that PACAP stimulates macrophage activity in arthritis and has distinct proinflammatory effects on phagocytes. The lack of increased inflammatory metabolic activity in the joints of PACAP^−/−^ mice shown by ^18^F-FDG–PET can be attributed to decreased macrophage activity (41).

The effects of PACAP on bone/cartilage metabolism and turnover have not previously been investigated in vivo under either normal or arthritic conditions. Our study provides substantial new evidence regarding its crucial role in bone pathophysiology. We demostrated that PACAP is a key regulator of bone turnover, particularly in inflammation. There are few, exclusively in vitro, data describing the ability of PACAP-38 to inhibit osteoblast precursors (24,25) and to diminish osteoclastogenesis (28). Taken together, the results of those studies and our present findings indicate that PACAP plays a crucial role in chondro- and osteogenesis by regulating bone formation and inhibiting pathologic osteophyte growth. Our finding of osteophyte formation in the late phase of disease indicates that this model has translational relevance regarding the degenerative complications of RA.

Synovial hyperplasia is a characteristic and widely investigated feature of RA. It is due to the formation of disinhibited fibroblast-like synoviocytes (FLS) that express NF-κB and secrete several inflammatory cytokines and adhesion molecules (interleukin-6, CCL-2, vascular cell adhesion molecule 1, intercellular adhesion molecule 1). Consequently, there is an influx and accumulation of inflammatory cells, which produce cytokines, chemokines, and proteases (matrix metalloproteinases, cathepsins, etc.), leading to cartilage and bone destruction. It was recently demonstrated that in the K/BxN model, FLS develop and exhibit behavior similar to that in human RA (49). This, taken together with the increased synovial hyperplasia in PACAP-deficient mice, suggests that PACAP might reduce FLS formation and exert a protective effect in chronic arthritis. This is also supported by the finding of pathologic bone neoformation in PACAP^−/−^ mice, which can be at least partially attributed to FLS-derived mediators.

The general limitations of experiments with knockout animals are that global gene deficiency can lead to potential phenotypic alterations during prenatal development and induce compensatory mechanisms. The possible effects of the flanking genes should also be taken into consideration. Discrepancies have been observed when comparing preclinical results obtained in knockout animals and clinical trial outcomes; for example, interferon-γ deficiency in mice was shown to induce arthritis, but clinical trials with interferon-γ resulted in only slight improvement in patients with RA (50).

In conclusion, PACAP deficiency has complex consequences with regard to several mechanisms related to RA. It decreases hyperemia, plasma leakage, and edema (most likely due to the lack of the potent vasodilating effect of PACAP) as well as functional impairment, and abolishes pain and sensitization. This in turn moderates the early migration of immune cells into the synovium and reduces metabolic activity. PACAP deficiency decreases early accumulation of neutrophils by slowing their extravasation from the vessels, but facilitates their function in the later phase. In addition, it decreases macrophage activity and ROS production and promotes inflammation-induced pathologic bone neoformation. We have provided experimental evidence of the important and complex regulatory function of PACAP. Identification of its targets and precise mechanisms might open future avenues for development of therapies aimed at both acute RA symptoms and chronic structural changes.

### Author Contributions

All authors were involved in drafting the article or revising it critically for important intellectual content, and all authors approved the final version to be published. Dr. Helyes had full access to all of the data in the study and takes responsibility for the integrity of the data and the accuracy of the data analysis.

**Study conception and design.** Botz, Németh, Szigeti, Horváth, Máthé, N. Kovács, Hashimoto, Reglődi, Szolcsányi, Pintér, Mócsai, Helyes.

**Acquisition of data.** Botz, Bölcskei, Kereskai, M. Kovács, Szigeti, Horváth, Máthé, N. Kovács, Helyes.

**Analysis and interpretation of data.** Botz, Bölcskei, Kereskai, Szigeti, Horváth, Máthé, N. Kovács, Hashimoto, Reglődi, Pintér, Helyes.

### Additional Disclosures

Authors Máthé and N. Kovács are employees of CROmed Ltd.
